# Cardiovascular Disease among Female Veterans of the 1991 Gulf War Era

**DOI:** 10.15436/2378-6841.19.2455

**Published:** 2019-03-29

**Authors:** Steven S. Coughlin, Vahe Heboyan, Kimberly Sullivan, Maxine Krengel, Col Candy Wilson, Stacey Iobst, Nancy Klimas

**Affiliations:** 1 Department of Population Health Sciences, Medical College of Georgia, Augusta University, Augusta, GA; 2 Research Service, Charlie Norwood Veterans Administration Medical Center, Augusta, GA; 3 Department of Interdisciplinary Health Sciences, College of Allied Health Sciences, Augusta University, Augusta, GA; 4 Boston University School of Public Health, Boston, MA; 5 VA Boston Healthcare System, Boston, MA; 6 Uniformed Services University Graduate School of Nursing, Bethesda, MD; 7 Henry M. Jackson Foundation at the Uniformed Services University Graduate School of Nursing, Bethesda, MD; 8 Miami VA Healthcare System, Miami, FL; 9 Institute for Neuro-Immune Medicine, Dr. Kiran C. Patel College of Osteopathic Medicine, Nova Southeastern University, Fort Lauderdale, FL

## Introduction

Recent clinical studies have identified exercise-induced transient postural tachycardia and abnormal heart-rate variability in patients with Gulf War Illness (GWI) (Rayhan et al. 2013; Garner et al. 2018; Blanchard et al. 2018). Altered heart rate variability may reflect autonomic dysfunction and atrophy in the cardio-regulatory regions of the brainstem (Rayhan et al. 2013). However, the long-term cardiovascular effects of abnormal autonomic nervous system functioning in patients with GWI are unknown (Blanchard et al. 2018). In additional clinical research studies, veterans with GWI have been found to have higher levels of cytokines such as interleukins (Coughlin 2017), which are inflammatory factors associated with increased risk of coronary heart disease and other chronic diseases (Lampert et al. 2006).

Using data from the Veterans Affairs (VA) Cooperative Studies Program 585 Gulf War Era Cohort and Biorepository (Khalil et al. 2018), this study examined the prevalence of cardiovascular disease among female veterans who served during the Gulf War or Gulf War Era. A total of 301 women veterans participated in the survey. Mean ages in 2016 were 53 years among women who were deployed and 54 years among women who were not deployed. About one-fifth of the participants were > 60 years of age. About three-quarters of the participants were white, 17–20% were Black or African American, and the remainder were American Indian/Alaska Native, Asian/Pacific Islander, or other race. About 6–8% of the participants were Hispanic or Latino. The majority of the participants had completed some college or received a college degree. In this sample, 12–13% of the participants were current cigarette smokers, 35% self-reported high blood pressure, and 40–41% self-reported high cholesterol. Compared to women veterans not deployed to the Gulf, deployed women veterans were not more likely to report cardiovascular disease (heart attack, coronary artery disease, congestive heart failure, stroke, or peripheral vascular disease). About 6.9% percent of the women who were deployed to the Gulf reported cardiovascular disease as compared to 11.2% of the women who were not deployed (odds ratio = 0.6, 95% confidence interval 0.3–1.4, P < 0.2).

Twenty-seven years after the 1990–1991 Gulf War, women veterans who were deployed to the Gulf continue to report similar levels of cardiovascular disease risk factors (cigarette smoking, high blood pressure, high cholesterol) as non-deployed women veterans who served during the Gulf War era. Women veterans deployed to the 1990–1991 Gulf War do not appear to be at increased risk of cardiovascular disease, although studies with longer duration of follow-up and larger sample sizes are needed.
